# Near-Fatal Acute Myocardial Infarction in a Young Patient With Occlusive Coronary Artery Disease

**DOI:** 10.7759/cureus.32012

**Published:** 2022-11-29

**Authors:** Carlson Sama, Muchi Ditah Chobufo, Ademola Ajibade, Joyce Foryoung, Melissa Roberts, Mark Greathouse

**Affiliations:** 1 Internal Medicine, West Virginia University School of Medicine, Morgantown , USA; 2 Cardiology, West Virginia University School of Medicine, Morgantown, USA; 3 Internal Medicine, West Virginia University School of Medicine, Morgantown, USA

**Keywords:** young patient, percutaneous coronary intervention, myocardial infarction, atherosclerosis, coronary artery disease

## Abstract

Acute myocardial infarction (AMI) due to obstructive coronary artery disease in young patients is an unusual event. Its clinical pattern somewhat differs from that of elderly patients, thus placing them at an increased risk of misdiagnosis, as this young population typically does not demonstrate the traditional risk factors associated with cardiovascular disease. We report the case of a 35-year-old man who presented with new-onset chest pain leading to cardiac arrest and was found to have 100% occlusion of the left anterior descending (LAD) coronary artery, which was successfully managed with the placement of a drug-eluting stent. We briefly reviewed the literature and noted that to reduce the risk of dramatic outcomes, it is imperative to include acute MI in the differential diagnosis of young patients presenting with chest pain, regardless of the presence or absence of any identifiable risk factor.

## Introduction

Coronary artery disease (CAD) remains a major cause of significant morbidity and mortality [1]. Although the process of atherosclerosis (the main driver of CAD) often commences in the early stages of life, the occurrence of symptomatic CAD and acute coronary syndrome (ACS) is often a chronic condition that predominately occurs after the sixth decade of life [1,2]. When compared to the elderly, the occurrence of acute myocardial infarction (AMI) in young people (45 years) is generally uncommon, and its aetiology is often multifactorial and varied, with several underlying medical and behavioral risk factors [3].

We herein discuss the case of a previously healthy 35-year-old man who presented with ST-segment elevation myocardial infarction and was found to have 100% occlusive CAD. This is generally an uncommon event when compared to the elderly, with few cases reported globally [4,5].

## Case presentation

The patient is a 35-year-old white male with a past medical history of anxiety. He has no prior cardiac or smoking history and does not use any routine medications. He was on duty as a firefighter worker when he suddenly developed a crushing, non-radiating, central chest pain associated with nausea, shortness of breath, and diaphoresis. He had never previously experienced chest pain at rest or during exertion. Due to the worsening of symptoms, emergency medical services (EMS) sought medical attention at the scene, and the cardiac monitor showed ST segment elevations in V1-V2, I/aVL, and ST depressions in leads II, III, and aVF. At that point in time, the patient went into ventricular fibrillation and became unresponsive and pulseless. Chest compressions were immediately started, and a one-time 200-J shock was delivered, which resulted in the return of spontaneous circulation. The patient was loaded with aspirin and amiodarone, and helicopter services were activated for prompt transfer to our academic percutaneous coronary intervention (PCI)-capable centre. En route, he developed a second episode of ventricular fibrillation whilst remaining conscious and promptly received a second 200-J shock with a return to normal sinus rhythm.

**Figure 1:**
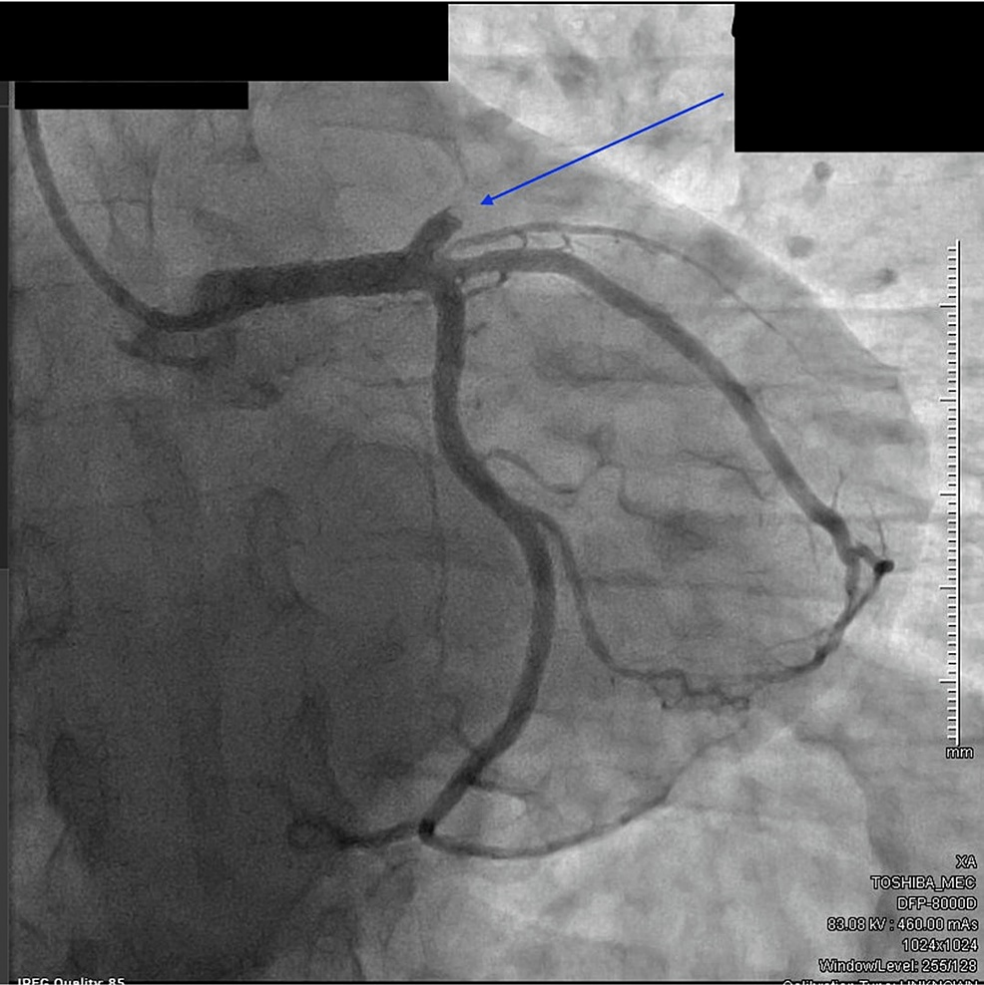
Pre-percutaneous coronary intervention coronary angiogram revealing 100% occlusion (blue arrow) of the left anterior descending coronary artery.

**Figure 2:**
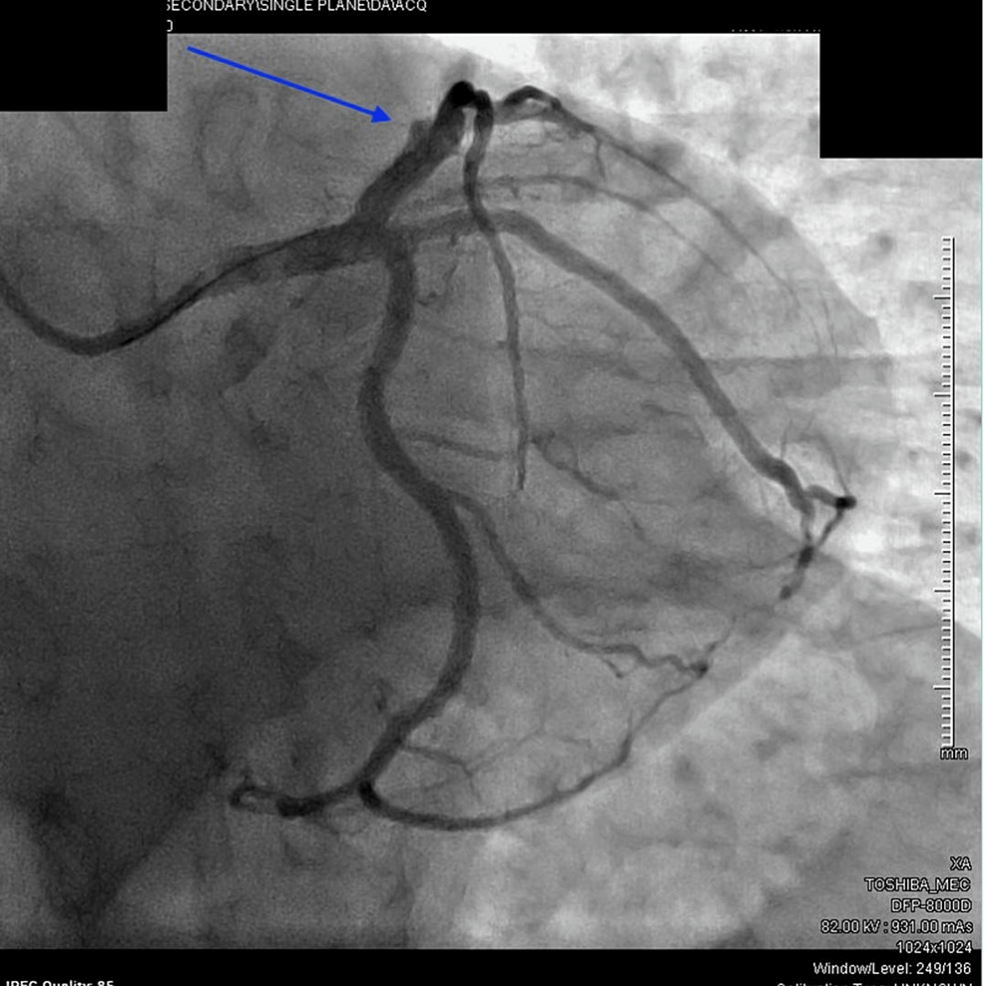
Post-intervention angiogram with normal flow in the left anterior descending artery (blue arrow).

**Figure 3:**
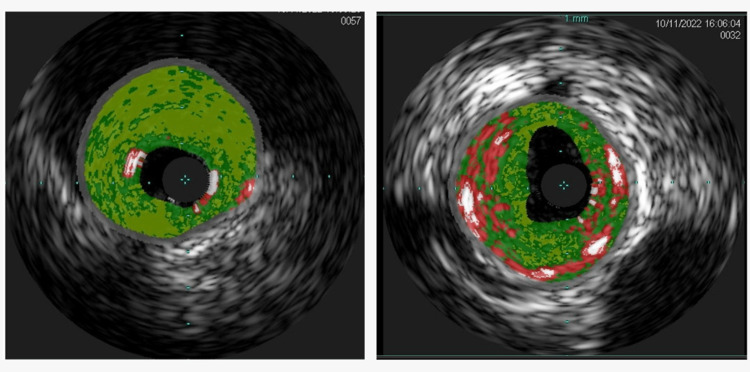
Intra-coronary (left anterior descending) ultrasound post-angiography revealing the underlying atherosclerotic disease.

**Figure 4:**
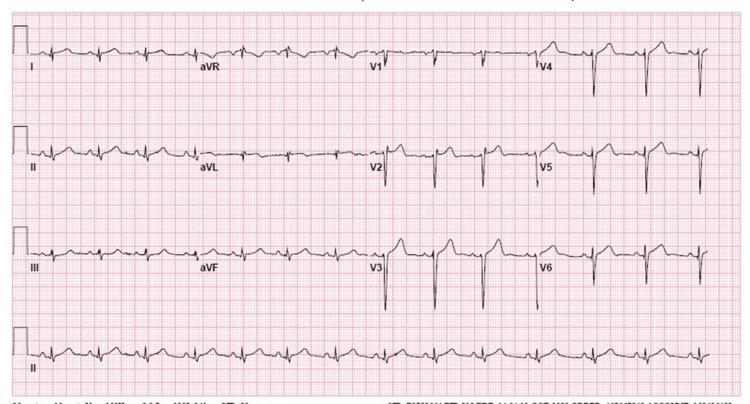
Post-percutaneous coronary intervention 12-lead electrocardiogram showing an incomplete right bundle branch block and anterior infarct.

Upon arrival at our facility, he was immediately taken for emergent left heart catheterisation, wherein the left anterior descending (LAD) artery was found to have a 100% occlusive proximal lesion (Figure 1) and the right coronary artery had a 20% stenosis in the mid-segment. He underwent a successful PCI to the proximal LAD coronary artery (culprit vessel) with a 3.5-mm × 22-mm Resolute Onyx drug-eluting stent, which resulted in thrombolysis in myocardial infarction (TIMI) grade 3 flow with 0 residual lesions (Figure 2). An intra-coronary ultrasound post-angiography revealed the underlying atherosclerotic disease (Figure 3). A 12-lead electrocardiogram post-PCI showed normal sinus rhythm with an incomplete right bundle branch block and an anterior infarct (Figure 4). It is important to note that the initial ECG on the field showing ST segment elevations in V1-V2, I/aVL, and ST depressions in leads II, III, and aVF was communicated by the EMS team directly to our Cath lab team via a telephone call but was not available for review by the time the patient arrived at our facility, potentially lost during the transportation phase. Given the acuity of the condition and prior confirmation of ECG findings by the EMS team and continual communication between teams during transportation, the patient was taken directly to the Cath lab without a repeat ECG upon arrival, and one was obtained only post-procedure.

His subsequent lipid panel was as follows: total cholesterol 216 mg/dl, high-density lipoproteins 52 mg/dl, low-density lipoproteins (LDLs) 152 mg/dl, very low-density lipoproteins 12 mg/dl, and triglycerides 59 mg/dl. His BMI was 27.25 kg/m^2^ and glycated haemoglobin levels were measured at 5.4%; the rest of his laboratory investigations were unremarkable. We believe that the main cause of his LAD obstruction was atherosclerotic plaque growth or thrombus formation secondary to plaque rupture or erosion.

Post-PCI, his left ventricular ejection fraction on a transthoracic echocardiogram was 35% with an akinetic mid-anterolateral and anteroseptal wall extending into the apex. He remained clinically stable and completely asymptomatic. He was started on aspirin, ticagrelor, atorvastatin, lisinopril, and metoprolol and discharged with no neurologic deficits. He was closely followed up in the clinic on day 6 post-discharge with optimisation of care and goal-directed medical therapy. Other than some residual chest soreness from prior chest compressions, he displayed no signs or symptoms suggestive of ongoing ACS or heart failure, reported being completely well in himself, and a cardiac life vest was in situ.

## Discussion

Although myocardial infarction (MI) predominantly affects the older population, data suggest that about 2-10% of all patients hospitalised with acute MI are <45 years of age [6] with some studies suggesting an increased incidence of CAD in very young people [7,8]. However, when MI does occur in this population, CAD is often one of several more common aetiologies [3]. We have just discussed the case of a young, healthy adult without any significant traditional cardiovascular risk factors presenting with advanced CAD complicated by a major adverse cardiac event (MACE). The presence of diabetes mellitus, an elevated Troponin T level, a lower left ventricular ejection fraction, and poor collaterals have been identified as some of the factors that predispose individuals to becoming hemodynamically unstable in an acute MI [9]. Compared to older patients, STEMI in young individuals generally has no ischaemic pre-conditioning and collateral vessel remodelling, thus progresses faster [10-12]. As reflected in our patients, this rapid progression places them at high risk of adverse outcomes, as the first manifestation of CAD is often an acute MI. This is opposed to the early warning ACS-like symptoms in older patients which offers a window of opportunity to intervene and reduce the risk of MACE [10-12]. Thus, in the current era of preventive cardiology, the study of CAD in the younger population is important.

Accelerated atherosclerosis is often the major causative factor of occlusive CAD, and though its prevalence in young adults is not well established, it is uncommon for CAD to cause STEMI in the young without disease drivers, which are generally rare. Homozygous familial hypercholesterolaemia, for example, affects ~1 in 1 million persons in the United States and appears to have the most consistent relationship with premature atherosclerosis and MI [13,14]. Though a combination of various other lipid fraction disorders is implicated [15], the main contributor to premature atherosclerosis and MI often involves mutations to the gene encoding the LDL receptor and often culminates in a dyslipidaemia clinical picture characterised by high serum cholesterol (primarily LDL fraction), xanthomas, xanthelasma, corneal arcus, and premature atherosclerosis [14,16]; all features which our patient did not display, thus further contributing to the case’s uniqueness.

Other risk factors for accelerated CAD in the young include smoking, therapeutic and illicit drug use, hypertension, hypercoagulable states, insulin resistance, obesity, inactivity, and stress [3,4,17]. It is important to recall that our patient was physically active, and other than a remote history of anxiety, which can potentiate MI [18], he did not display any of the above risk factors. Though he only had a moderate degree of dyslipidaemia and no overt clinical features of lipid disorders, we hypothesised that this might have played a role in his major adverse cardiac event, as a study series of 67 patients found that in the absence of other obvious risk factors for MI, hypercholesterolaemia is the most important one [4,14]. Furthermore, family history is particularly important and has a strong correlation, with up to a 10-fold increased risk of developing CAD in siblings of a young patient with MI [14,19]. However, our patient was adopted and has no knowledge of any family history. It is plausible that familial genetic factors might have in part played a contributory role to his course, however, the basis of this genetic susceptibility towards atherosclerosis in the young remains unclear as the genetic research continues.

## Conclusions

The occurrence of total LAD obstruction due to atherosclerotic plaque growth or thrombus formation secondary to plaque rupture resulting in a STEMI in a 35-year-old patient is an unusual event when compared to elderly patients. The lack of collaterals and ischaemic pre-conditioning places them at increased risk for adverse outcomes such as recurrent ventricular fibrillation. This clinical vignette suggests a high index of suspicion must be maintained in young patients presenting with chest pain even without known risk factors if we are to reduce the significant morbidity and mortality associated with major adverse cardiac events in this population. In addition, the role of risk factor modification and secondary prevention cannot be overemphasized.
